# Synergistically improved cardiovascular outcomes in type 2 diabetes mellitus patients with combined treatment of SGLT-2 inhibitors and pioglitazone

**DOI:** 10.3389/fendo.2024.1420485

**Published:** 2024-08-30

**Authors:** Yan-Rong Li, Chih-Ching Wang, Chi-Hung Liu, Chieh-Li Yen, Victor Chien-Chia Wu, Evelyn Jou-Chen Huang, Ching-Yu Lee, Ching-Chung Hsiao

**Affiliations:** ^1^ Division of Endocrinology and Metabolism, Department of Internal Medicine, Linkou Chang Gung Memorial Hospital, Taoyuan, Taiwan; ^2^ College of Medicine, Chang Gung University, Taoyuan, Taiwan; ^3^ College of Medicine, National Tsing Hua University, Hsinchu, Taiwan; ^4^ Stroke Center and Department of Neurology, Linkou Chang Gung Memorial Hospital, Taoyuan, Taiwan; ^5^ Kidney Research Center, Department of Nephrology, Linkou Chang Gung Memorial Hospital, Taoyuan, Taiwan; ^6^ Division of Cardiology, Linkou Chang Gung Memorial Hospital, Taoyuan, Taiwan; ^7^ Department of Ophthalmology, Taipei Medical University Hospital, Taipei, Taiwan; ^8^ Department of Ophthalmology, School of Medicine, College of Medicine, Taipei Medical University, Taipei, Taiwan; ^9^ Department of Orthopedics, Taipei Medical University Hospital, Taipei, Taiwan; ^10^ Department of Orthopedics, School of Medicine, College of Medicine, Taipei Medical University, Taipei, Taiwan; ^11^ International PhD Program for Cell Therapy and Regeneration Medicine, College of Medicine, Taipei Medical University, Taipei, Taiwan; ^12^ Department of Nephrology, New Taipei Municipal TuCheng Hospital, New Taipei City, Taiwan

**Keywords:** type 2 diabetes mellitus, sodium-glucose co-transporter-2 inhibitors (SGLT2i), pioglitazone, cardiovascular outcomes, heart failure

## Abstract

**Background:**

Sodium-glucose co-transporter-2 inhibitors (SGLT2i) have cardiovascular (CV) benefits, particularly in reducing the risk of heart failure (HF). Pioglitazone (Pio) has shown potential in decreasing the risks of recurrent stroke, non-fatal myocardial infarction (MI), and all-cause mortality but increasing risks of HF. Our study aimed to examine the synergistic effects on CV outcomes in patients with type 2 diabetes mellitus (T2DM) who received the combined treatment of SGLT2i and Pio.

**Materials and methods:**

A total of 117,850 patients with T2DM and without a history of HF were selected as the observational study cohort from the Chang Gung Research Database (CGRD) in Taiwan between January 1, 2016, and December 31, 2019. The primary composite outcome was 4-point major adverse CV events (4P-MACE), including CV death, non-fatal MI, non-fatal ischemic stroke, and hospitalization for HF. The study was divided into four groups: a combined treatment group in which SGLT2i and Pio were used, two individual groups in which SGLT2i or Pio was used separately, and a reference group (non-study drugs).

**Results:**

Combined treatment of SGLT2i and Pio had the lowest risk of 4P-MACE (adjusted hazard ratio [aHR], 0.66; 95% confidence interval [CI], 0.54–0.80) compared with the reference group after a mean follow-up of 2.2 years. There was no significant difference in risks of hospitalization for HF (adjusted subdistribution hazard ratio, 0.73; 95% CI, 0.49–1.07) compared with the reference group.

**Conclusions:**

In T2DM patients without HF, the combined treatment with SGLT2i and Pio may synergistically provide CV benefits without increasing risks of HF.

## Introduction

Type 2 diabetes mellitus (T2DM) is considered a coronary heart disease equivalent (1) and is associated with approximately a 2-fold increased risk of stroke compared to individuals without T2DM (2, 3). Additionally, the mortality rate among T2DM patients with heart failure (HF) has been reported to be as high as 32.7% per year (4). Therefore, the first priority in treating T2DM patients is to decrease the risks of cardiovascular (CV) complications and HF.

Sodium-glucose co-transporter-2 inhibitors (SGLT2i) are a class of glucose-lowering agents that increase urinary glucose excretion independently of insulin action. Several landmark randomized clinical trials of SGLT2i, including empagliflozin, canagliflozin, and dapagliflozin, have demonstrated not only reductions in blood glucose levels but also significant CV protection, particularly in reducing the risk of heart failure (5–9). However, in the EMPA-REG trial, the risk of stroke after treatment with empagliflozin increased nonsignificantly (hazard ratio [HR], 1.18; 95% confidence interval [CI], 0.89–1.56; P=0.26) (5). As a result, for stroke prevention in T2DM patients, besides injection therapy with glucagon-like peptide-1 receptor agonists (GLP-1 RAs), which have been shown to reduce the risk of stroke in some clinical trials (10), another oral glucose-lowering agent should be considered in daily practice because some patients may not accept or tolerate injection therapy.

Pioglitazone (Pio), a thiazolidinedione (TZD) with vascular protection and the ability to ameliorate atherosclerosis progression due to its potent effect as a peroxisome proliferator-activated receptor-γ (PPAR-γ) agonist, may reduce recurrent stroke in patients with T2DM or insulin resistance (11, 12). However, increased risks of HF have been noted (13). Furthermore, for T2DM patients with high CV risks, the PROactive study revealed that Pio may decrease all-cause mortality, non-fatal myocardial infarction (MI), and stroke by 16% (14). Given these considerations, combined treatment with SGLT2i and Pio is logical because these two anti-hyperglycemic agents offer individual CV benefits, and SGLT2i can mitigate the risk of HF associated with Pio use. Therefore, the aim of our real-world cohort study was to investigate whether combined treatment with SGLT2i and Pio in T2DM patients is associated with better CV outcomes.

## Materials and methods

### Data source

This multi-institutional cohort study utilized retrospective data from the Chang Gung Research Database (CGRD), spanning from 2001 to 2019. The CGRD is Taiwan’s largest healthcare provider database, comprising information from four tertiary academic centers and three teaching hospitals, with nationwide coverage representing approximately 6% of the population (15). Data generated before 2015 were identified and registered using the International Classification of Diseases, Ninth Revision, Clinical Modification (ICD-9-CM), while data from 2016 onwards utilized both ICD-9-CM and the International Classification of Diseases, Tenth Revision, Clinical Modification (ICD-10-CM). Previous studies utilizing CGRD data have demonstrated the validity of assessment and treatment outcomes (16–19). The study protocol was approved by the institutional review board of Chang Gung Medical Foundation (IRB No: 202100656B0), and informed consent was waived as patient information had been de-identified in the CGRD.

### Patients selection and study design

**Figure 1:**
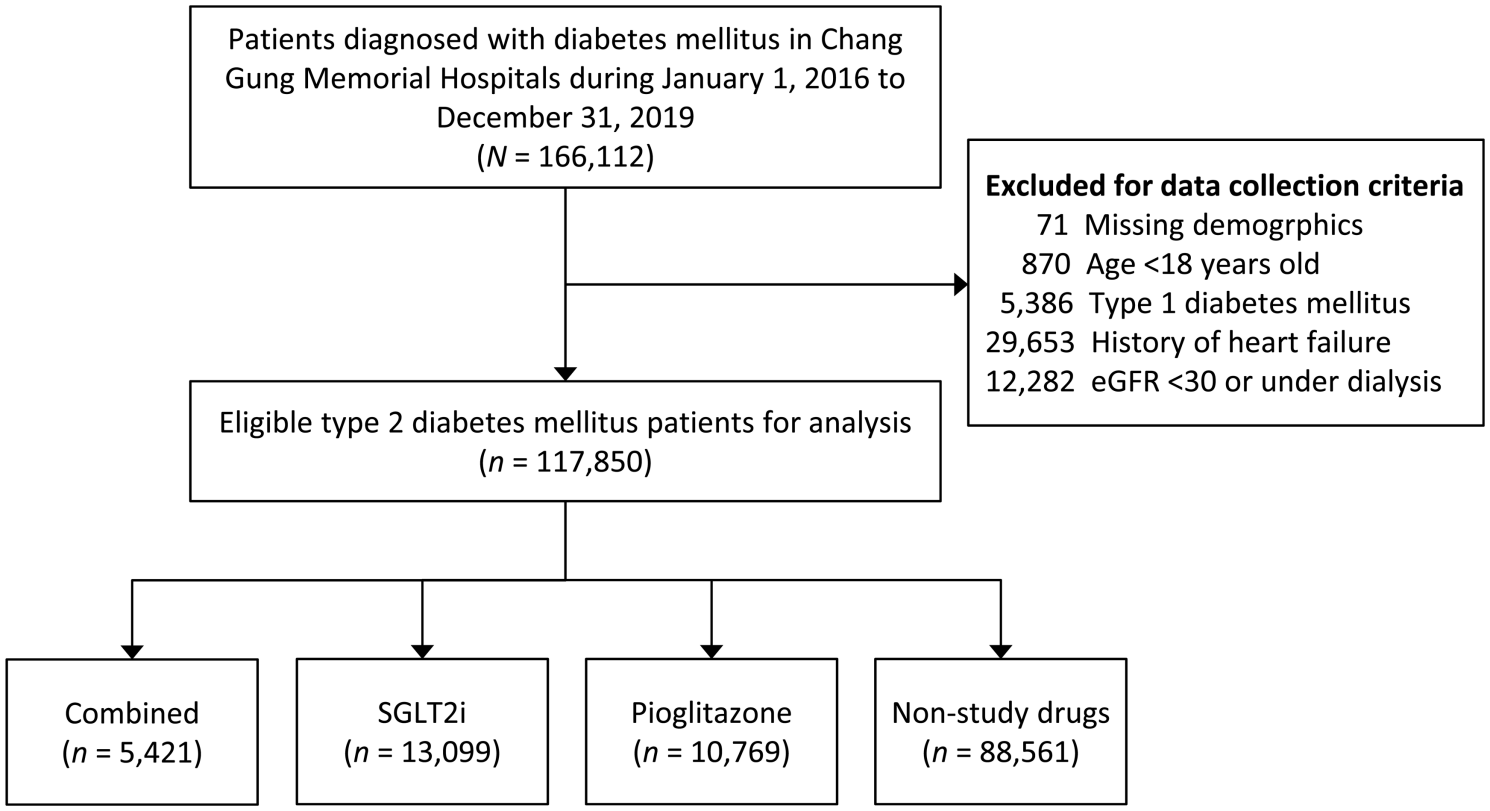
Flowchart for the inclusion and exclusion of the study patients.

**Figure 1** illustrates the process used to select participants for the study cohorts. We conducted a search of electronic medical records from the CGRD covering the period between January 1, 2016, and December 31, 2019, to identify patients with a diagnosis of DM (N=166,112). Patients with missing demographics (i.e., age and sex) and those under the age of 18 were excluded. Since our objective was to investigate the effects of SGLT2i and Pio in T2DM patients, individuals with T1DM (N=5,386) were also excluded. To assess the association between the study drugs and heart failure and because Pio should be avoided for patients with HF, patients with a history of HF (N=29,653) were excluded from the present study. Since the SGLT2i had uncertain effects and was not certified by Taiwan National Health Insurance for patients with a glomerular filtration rate <30ml/min/1.72m2 until December 31, 2019, patients with advanced CKD (eGFR <30ml/min/1.72m2) or those receiving dialysis (N=12,282) were excluded. The diagnosis of T2DM was defined as having at least two outpatient claims or one inpatient claim with ICD-9-CM code = 250 or ICD-10-CM code = E11, and the use of at least one kind of anti-diabetic agent, such as metformin, sulfonylurea, glinides, pioglitazone, acarbose, dipeptidylpeptidase-4 inhibitors, glucagon-like peptide-1 receptor agonist, SGLT2i, or insulin. Ultimately, a total of 117,850 patients with T2DM were analyzed in the study cohort (**Figure 1**).

### Exposure to the study drugs

This study was divided into four groups according to the exposure status of SGLT2i and Pio to assess the CV outcomes. The group 1 consisted of T2DM patients receiving both SGLT2i and Pio (combined); the group 2 consisted of T2DM patients receiving SGLT2i; the group 3 consisted of T2DM patients receiving Pio; the group 4 (the reference group) consisted of those receiving non-study drugs. We extracted medication data from outpatient claims or pharmacy refills for chronic illnesses, defining SGLT2i users or Pio users if the medication was prescribed for more than three months. Since the SGLT2i was initially introduced in Taiwan in 2015 and became available at our hospital since 2016, we defined the index date as three months after the date of the first prescription of SGLT2i and/or Pio in the groups 1-3, and the index date in group 4 (the reference group) as three months after the date of the first prescription of other anti-hyperglycemic agents between January 1, 2016, and December 31, 2019.

### Covariates

The covariates analyzed in this study encompass demographics, comorbidities, baseline vital signs, concomitant medications, and baseline laboratory data. Demographic factors include age, sex, body mass index (BMI), hospital level (medical center or non-medical center), and duration of diabetes mellitus (DM), with the earliest DM diagnosis traced back to 2001. Comorbidities assessed comprise stroke (hospitalization), MI (hospitalization), atrial fibrillation, coronary artery disease (CAD), peripheral artery disease (PAD), hypertension, dyslipidemia, chronic kidney disease (CKD), chronic obstructive pulmonary disease, gout, venous thromboembolism, autoimmune disease, and history of malignancy.

Baseline vital signs encompass systolic and diastolic blood pressure. Concomitant medications were categorized into anti-hypertensive agents, anti-diabetic agents, and others. Baseline laboratory values include glycohemoglobin (HbA1c), low-density lipoprotein cholesterol (LDL-C), high-density lipoprotein cholesterol (HDL-C), serum creatinine, estimated glomerular filtration rate (eGFR), and alanine aminotransferase (ALT). Previous validation studies have confirmed the accuracy of diagnostic codes for these events and comorbidities (20, 21).

Baseline medication usage was identified within the same period as the study drugs. BMI, vital signs, and baseline laboratory results were derived from the most recent records within three months preceding the index date.

### Outcomes measurement

The primary outcome of this study was defined as the composite of 4-point major adverse CV events (4P-MACE), which includes CV death, non-fatal MI, non-fatal ischemic stroke, and hospitalization for HF. Secondary outcomes aimed to investigate all-cause mortality and adverse events, including diabetic ketoacidosis (DKA) or hyperglycemic hyperosmolar state (HHS), hypoglycemia with laboratory glucose <55mg/dL, newly diagnosed malignancy, newly diagnosed atrial fibrillation, and acute hepatitis.

To ascertain mortality data, the date and cause of death were linked with the Taiwan Death Registry database. CV death was defined according to the Standardized Definitions for Cardiovascular and Stroke Endpoint Events in Clinical Trials established by the United States Food and Drug Administration (FDA). The occurrences of MI, stroke, and hospitalization for HF were identified based on the primary discharge diagnosis. DKA, HHS, and acute hepatitis were identified using discharge diagnosis codes or diagnoses from the emergency department.

The follow-up duration extended from the index date until death, the last visit in hospitals, or the conclusion of the follow-up period (December 31, 2019), whichever came first.

### Statistical analysis

The demographics and baseline characteristics of patients for the four study groups were summarized, although significance tests between groups were not conducted due to the relatively large sample size (nearly 120,000 people). Instead, the maximum absolute standardized difference (MASD) was used to illustrate the largest difference among the six pairwise comparisons. Due to the relatively low incidence of several primary outcomes (CV death, non-fatal MI, hospitalization for HF, etc.), multivariable covariates adjustment may lead to overfitting. Instead of traditional multivariable adjustment, we adopted an adjustment using multiple propensity scores. Initially, a multivariable multinomial logistic model was constructed, considering the study groups (4 categories) as outcome variables and including all baseline characteristics (but not including the outcomes of interest and the duration of follow-up) as covariates with a forced entry. The index date was also introduced into the model to enable the duration of follow-up to be potentially equal among the study groups. Consequently, 4 estimated probabilities (referred to as propensity scores) were generated for each individual regarding membership in a specific group. Adjusting any three out of the four propensity scores could minimize group differences associated with the baseline characteristics (22).

We compared the risks of 4P-MACE and fatal events (i.e., CV death and all-cause death) among groups using the Cox proportional hazard model. The incidence of other outcomes among groups was compared using the Fine and Gray subdistribution hazard model, which considered death as a competing risk. We were further particularly interested in comparing the risk of 4P-MACE between the combined drugs group (group 1) and the non-study drugs group (group 4, the reference group) stratified by several subgroup variables, including age (<65 vs. ≥65 years), sex, durations of diabetes (<5, 5-9.9, and ≥10 years), previous stroke, previous MI, baseline HbA1c value (<7, 7-9, and >9%) and baseline renal function (eGFR 30-60, 61-90 and >90 ml/min/1.72m^2^). Statistics analyses were performed using SAS (version 9.4; SAS Institute, Cary, NC, USA). A two-sided *P* value of <0.05 was considered to be significant.

## Results

### Study patients

Between 1 January 2016 and 31 December 2019, a total of 166,112 patients with a diagnosis of DM were recorded in the CGRD. After applying exclusion criteria, a total of 117,850 patients with T2DM were confirmed as eligible for the analysis. Among these, the group 1 (combined use with SGLT2i and Pio) had 5,421 patients, the group 2 (SGLT2i) had 13,099 patients, the group 3 (Pio) had 10,769 patients, and the group 4 (non-study drugs, the reference group) had 88,561 patients (**Figure 1**).

The baseline characteristics of patients were detailed in **Table 1**. Briefly, patients who took SGLT2i (group 1 and group 2) were younger than those who did not take SGLT2i (group 3 and group 4). Moreover, patients who took study drugs (group 1, group 2 and group 3) tended to have poor baseline condition, including higher BMI values, more comorbidities (e.g., CAD, dyslipidemia, and CKD) and polypharmacy when comparing to the group 4. Poor baseline renal function was observed in the group 3 (Pio), while slightly better liver function was noted (**Table 1**).

**Table 1:** Demographics and baseline characteristics of the study patients according to the use of SGLT2i and pioglitazone.

Variable	Available number	Group 1 (Combined) (*n* = 5,421)	Group 2 (SGLT2i) (*n* = 13,099)	Group 3 (Pio) (*n* = 10,769)	Group 4 (Non-study drugs) (*n* = 88,561)	MASD
Demographics						
Age, year	117,850	59.5 ± 11.0	58.9 ± 11.9	63.5 ± 11.7	63.3 ± 12.9	0.38
Male sex	117,850	3,388 (62.5)	7,377 (56.3)	6,108 (56.7)	47,758 (53.9)	0.17
Body mass index, kg/m^2^	111,116	27.9 ± 4.7	27.8 ± 4.6	26.8 ± 4.3	26.1 ± 4.4	0.40
Hospital level	117,850					0.25
Medical center		2,882 (53.2)	5,615 (42.9)	5,936 (55.1)	46,150 (52.1)	
Regional/district hospital		2,539 (46.8)	7,484 (57.1)	4,833 (44.9)	42,411 (47.9)	
Diabetes duration, year	117,850	8.6 ± 5.4	7.2 ± 5.4	8.1 ± 5.4	4.7 ± 5.1	0.75
Comorbidity						
Previous stroke	117,850	359 (6.6)	721 (5.5)	1,059 (9.8)	8,498 (9.6)	0.15
Previous myocardial infarction	117,850	223 (4.1)	585 (4.5)	223 (2.1)	1,864 (2.1)	0.16
Coronary artery disease	117,850	1,192 (22.0)	2,624 (20.0)	1,752 (16.3)	12,829 (14.5)	0.21
Atrial fibrillation	117,850	112 (2.1)	271 (2.1)	180 (1.7)	2,091 (2.4)	0.05
Peripheral artery disease	117,850	167 (3.1)	417 (3.2)	405 (3.8)	2,683 (3.0)	0.04
Hypertension	117,850	3,855 (71.1)	8,916 (68.1)	7,468 (69.3)	54,747 (61.8)	0.19
Dyslipidemia	117,850	4,354 (80.3)	9,837 (75.1)	7,872 (73.1)	50,644 (57.2)	0.48
Chronic kidney disease	117,850	2,476 (45.7)	4,701 (35.9)	4,697 (43.6)	23,464 (26.5)	0.42
Chronic obstructive pulmonary disease	117,850	529 (9.8)	1,111 (8.5)	1,129 (10.5)	8,751 (9.9)	0.07
Gout	117,850	565 (10.4)	1,368 (10.4)	1,010 (9.4)	8,271 (9.3)	0.04
Venous thromboembolism	117,850	42 (0.8)	125 (1.0)	91 (0.8)	694 (0.8)	0.02
Autoimmune disease	117,850	87 (1.6)	240 (1.8)	172 (1.6)	1,614 (1.8)	0.02
History of malignancy	117,850	421 (7.8)	1,087 (8.3)	971 (9.0)	10,388 (11.7)	0.13
Baseline vital signs						
Systolic blood pressure, mmHg	109,687	139.4 ± 19.4	138.8 ± 19.1	140.5 ± 20.1	139.1 ± 20.9	0.09
Diastolic blood pressure, mmHg	109,654	78.1 ± 11.6	78.1 ± 11.3	77.0 ± 11.5	77.6 ± 12.1	0.10
Anti-hypertensive agents						
ACEI/ARB	117,850	3,181 (58.7)	7,356 (56.2)	5,962 (55.4)	39,153 (44.2)	0.29
Calcium-channel blockers	117,850	1,205 (22.2)	3,211 (24.5)	2,763 (25.7)	23,029 (26.0)	0.09
Alpha-blocker	117,850	417 (7.7)	999 (7.6)	1,013 (9.4)	8,161 (9.2)	0.06
Beta-blocker	117,850	1,390 (25.6)	3,155 (24.1)	2,095 (19.5)	15,505 (17.5)	0.21
Thiazide	117,850	157 (2.9)	362 (2.8)	305 (2.8)	1,891 (2.1)	0.05
Anti-diabetic agents						
Biguanide (Metformin)	117,850	5,217 (96.2)	11,926 (91.0)	9,866 (91.6)	70,832 (80.0)	0.43
Sulfonylurea	117,850	4,264 (78.7)	8,531 (65.1)	8,222 (76.3)	35,437 (40.0)	0.77
Glinide	117,850	140 (2.6)	341 (2.6)	428 (4.0)	2,745 (3.1)	0.08
DPP-4 inhibitor	117,850	3,792 (70.0)	8,014 (61.2)	7,621 (70.8)	32,975 (37.2)	0.68
GLP-1 RA	117,850	149 (2.7)	335 (2.6)	248 (2.3)	302 (0.3)	0.26
Insulin	117,850	495 (9.1)	1,834 (14.0)	1,101 (10.2)	15,147 (17.1)	0.22
Alpha glucosidase inhibitors	117,850	1,358 (25.1)	2,202 (16.8)	2,121 (19.7)	7,290 (8.2)	0.54
Other medications						
Aspirin	117,850	1,723 (31.8)	3,322 (25.4)	3,073 (28.5)	20,256 (22.9)	0.21
Clopidogrel	117,850	293 (5.4)	774 (5.9)	564 (5.2)	4,357 (4.9)	0.05
Cilostazol	117,850	70 (1.3)	194 (1.5)	170 (1.6)	1,219 (1.4)	0.02
Statin	117,850	3,972 (73.3)	8,675 (66.2)	6,972 (64.7)	40,857 (46.1)	0.54
Fibrate	117,850	472 (8.7)	1,195 (9.1)	777 (7.2)	4,661 (5.3)	0.16
Ezetimibe	117,850	700 (12.9)	1,418 (10.8)	1,002 (9.3)	5,130 (5.8)	0.28
NSAIDs or COX-2i	117,850	944 (17.4)	2,657 (20.3)	2,218 (20.6)	22,466 (25.4)	0.19
Steroid	117,850	166 (3.1)	483 (3.7)	440 (4.1)	4,993 (5.6)	0.12
Baseline laboratory data						
Glycated hemoglobin, %	111,268	8.8 ± 1.5	8.7 ± 1.7	8.3 ± 1.7	7.9 ± 1.9	0.52
Low-density lipoprotein, mg/dL	105,656	81.1 ± 53.1	79.6 ± 58.2	74.2 ± 50.8	74.9 ± 56.5	0.13
High-density lipoprotein, mg/dL	101,984	46.2 ± 12.3	43.8 ± 11.1	46.6 ± 12.3	45.5 ± 12.2	0.24
Creatinine, mg/dL	114,838	0.88 ± 0.26	0.85 ± 0.27	0.94 ± 0.33	0.88 ± 0.30	0.27
eGFR, ml/min/1.73m^2^	114,838	92.1 ± 27.7	95.2 ± 30.3	86.9 ± 32.7	92.4 ± 33.4	0.26
Alanine amino transferase, U/L	106,956	30.8 ± 22.4	35.8 ± 27.0	28.5 ± 22.1	31.3 ± 25.2	0.30
Follow-up year	117,850	1.9 ± 1.1	1.9 ± 1.1	2.4 ± 1.4	2.5 ± 1.4	0.43

**Table:** 

Disease	ICD-9-CM	ICD-10-CM
Diabetes mellitus	250.xx	E08-E13
Type 1 diabetes mellitus	250.01, 250.03,250.11, 250.13,250.21, 250.23,250.31, 250.33,250.41, 250.43,250.51, 250.53,250.61, 250.63,250.71, 250.73,250.81, 250.83,250.91, 250.93	E10
Heart failure	428.xx	I50
Stroke	430.xx–437.xx	I60-I62, I66, I65.1, I65.0, I65.8, I65.9, I63.6, I63.8, I63.9, G45.0, G45.8, G45.1, G45.2, G46.0, G46.1, G46.2, G45.9, G45.4, G46.3, G46.4, G46.5, G46.6, G46.7, G46.8, I67.0, I67.1, I67.2, I67.4, I67.5, I67.6, I67.7, I67.9, I68.0, I68.2, I68.8
Previous myocardial infarction	410.xx, 412.xx	I21-I22
Coronary artery disease	410.xx-414.xx	I20-I24
Atrial fibrillation	427.3x	I48
Peripheral arterial disease	440.xx, 441.xx, 443.xx, 444.0x, 444.8x, 447.8x, 447.9x, 093.0, 437.3, 444.22, 447.1, 557.1, 557.9, V434	I70, I71, I73, I75, I771, I790, I791, I792, I773, I779, I798, K551, K558, K559, Z958, Z959, I743, I744, I745, I748, I740, I7789
Hypertension	401.xx-405.xx	I10-I15, N262
Dyslipidemia	272.xx	E77, E780, E781, E782, E783, E784, E785, E786, E881, E753, E755, E882, E756, E789, E7521, E7522, E7524, E7130, E7879, E7881, E7889, E8889, E7870
Chronic kidney disease	580.xx-589.xx, 403.xx-404.xx, 016.0x, 095.4x, 236.9x, 250.4x, 274.1x, 442.1x, 447.3x, 440.1x, 572.4x, 642.1x, 646.2x, 753.1x, 283.11, 403.01, 404.02, 446.21	A1811, D593, E102, E112, E132, I12, I13, K767, M103, M310, N00, N01, N02, N03, N04, N05, N06, N07, N08, N14, N150, N158, N159, N16, N171, N172, N18, N19, N200, N25, N261, N269, N27, Q61
Dialysis	585.xx with dialysis treatment	N18 with dialysis treatment
Chronic obstructive pulmonary disease	491.xx, 492.xx, 496.xx	J41-J44
Gout	274.xx	M10, M1A
Pulmonary embolism	415.1x	I26
Deep vein thrombosis	453.xx	I81,I82
Autoimmune disease	710.0, 710.1, 714.0, 710.4, 710.3, 446.0, 446.2, 446.4, 446.5, 443.1, 446.7, 136.1, 694.4, 710.2, 555.xx, 556.xx	L10, K51, I731, M057, M058, M059, M060, M061, M062, M063, M068, M069, M300, M301, M302, M308, M310, M314, M315, M316, M317, M320, M328, M329, M340, M341, M342, M349, M352, M360, K5000, K5010, K5080, K5090, M3210, M3219, M3300, M3309, M3310, M3319, M3320, M3329, M3390, M3399, M3489, M3500, M3501, M3509, K50011, K50018, K50019, K50111, K50118, K50119, K50811, K50818, K50819, K50911, K50918, K50919
Malignancy	140.xx–208.xx	C00-C96
Cardiovascular death	390.xx – 459.xx, 785.5x	I00-I99, R570, R579
DKA or HHS	250.1x, 250.2x, 250.3x	E10.10, E10.11, E10.65, E11.65, E13.10, E13.11, E13.65
Acute hepatitis	277.4, 570, 572.8, 573.3, 573.8, 576.8, 782.4	B15, B16, B17, K72.0

SGLT2i, sodium-glucose co-transporter-2 inhibitors; MASD, maximum absolute standardized difference; ACEI/ARB, angiotensin-converting enzyme inhibitors/angiotensin receptor blocker; DPP-4, dipeptidyl peptidase 4; GLP1-RA, glucagon like peptide-1 receptor agonist; NSAIDs, non-steroidal anti-inflammatory drugs; COX-2i, cyclooxygenase-2 inhibitors; eGFR, estimated glomerular filtration rate; DKA, diabetic ketoacidosis; HHS, hyperosmolarhyperglycemia; Data were presented as frequency (percentage) or mean ± standard deviation.

### Effects of SGLT2i and Pio on 4P-MACE in T2DM patients

**Figure 2:**
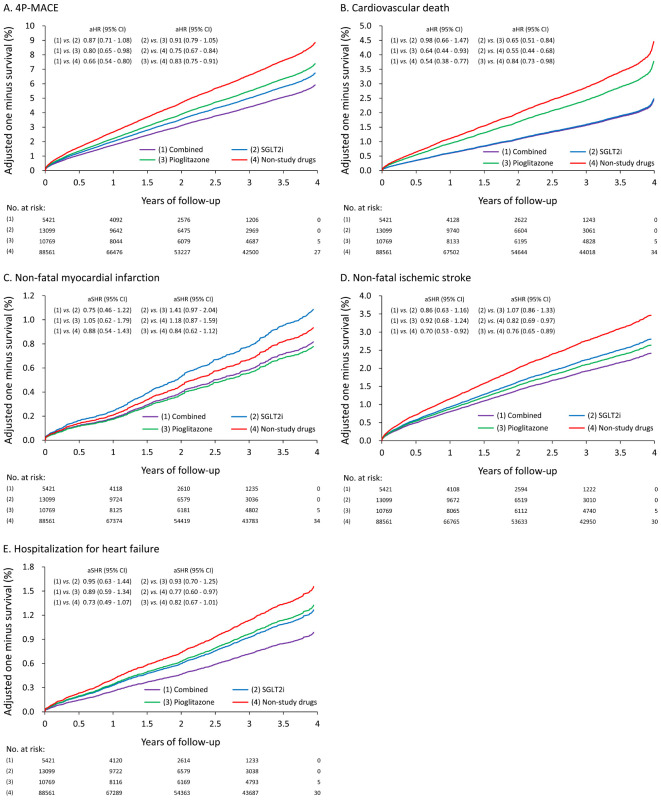
The adjusted/fitted (by multiple propensity scores) one minus survival rates of T2DM patients according to the use of SGLT2i and Pio: **(A)** 4P-MACE **(B)** Cardiovascular death **(C)** Non-fatal myocardial infarction **(D)** Non-fatal ischemic stroke **(E)** Hospitalization for heart failure.

During a mean follow-up of 2.2 years (standard deviation: 1.4 years), the results revealed that both the combined use and individual use of SGLT2i or Pio were associated with a lower risk of 4P-MACE compared to non-study drugs (combined: adjusted hazard ratio [aHR], 0.66; 95% CI, 0.54–0.80; SGLT2i: aHR, 0.75; 95% CI, 0.67–0.84; Pio: aHR: 0.83, 95% CI, 0.75–0.91, respectively). Notably, the combined use of SGLT2i and Pio demonstrated the lowest risk of 4P-MACE compared to non-study drugs (aHR, 0.66; 95% CI, 0.54–0.80) (**Table 2**; **Figure 2A**).

**Table 2:** Primary cardiovascular outcomes of patients according to the use of SGLT2i and pioglitazone.

Outcome/group	Incidence† (95% CI)	Adjusted HR or SHR (95% CI) (column vs row)			Adjusted *P* value of trend test
		Combined	SGLT2i	Pioglitazone	
4P-MACE					<0.001
Non-study drugs	25.2 (24.5–25.9)	0.66 (0.54–0.80)*	0.75 (0.67–0.84)*	0.83 (0.75–0.91)*	
Pioglitazone	19.0 (17.3–20.7)	0.80 (0.65–0.98)*	0.91 (0.79–1.05)		
SGLT2i	14.3 (12.8–15.8)	0.87 (0.71–1.08)			
Combined	12.3 (10.1–14.4)	–	–	–	
Cardiovascular death					<0.001
Non-study drugs	11.7 (11.2–12.1)	0.54 (0.38–0.77)*	0.55 (0.44–0.68)*	0.84 (0.73–0.98)*	
Pioglitazone	7.8 (6.7–8.8)	0.64 (0.44–0.93)*	0.65 (0.51–0.84)*		
SGLT2i	3.6 (2.9–4.4)	0.98 (0.66–1.47)			
Combined	3.3 (2.2–4.4)	–	–	–	
Non-fatal myocardial infarction					0.923
Non-study drugs	2.3 (2.1–2.5)	0.88 (0.54–1.43)	1.18 (0.87–1.59)	0.84 (0.62–1.12)	
Pioglitazone	2.1 (1.5–2.6)	1.05 (0.62–1.79)	1.41 (0.97–2.04)		
SGLT2i	2.7 (2.1–3.4)	0.75 (0.46–1.22)			
Combined	2.1 (1.2–3.0)	–	–	–	
Non-fatal ischemic stroke					<0.001
Non-study drugs	9.8 (9.4–10.3)	0.70 (0.53–0.92)*	0.82 (0.69–0.97)*	0.76 (0.65–0.89)*	
Pioglitazone	7.6 (6.5–8.6)	0.92 (0.68–1.24)	1.07 (0.86–1.33)		
SGLT2i	6.8 (5.8–7.9)	0.86 (0.63–1.16)			
Combined	6.0 (4.5–7.5)	–	–	–	
Hospitalization for heart failure					0.021
Non-study drugs	4.1 (3.8–4.3)	0.73 (0.49–1.07)	0.77 (0.60–0.97)*	0.82 (0.67–1.01)	
Pioglitazone	3.4 (2.7–4.2)	0.89 (0.59–1.34)	0.93 (0.70–1.25)		
SGLT2i	2.7 (2.1–3.4)	0.95 (0.63–1.44)			
Combined	2.1 (1.2–3.0)	–	–	–	

4P-MACE, 4-point major adverse cardiac events; SGLT2i, sodium-glucose co-transporter-2 inhibitors; HR, hazard ratio; SHR, sub-distribution hazard ratio; CI, confidence interval.

†Number of events per 1000 person-years.

Anyone of the cardiovascular death, non-fatal myocardial infarction and non-fatal stroke.

*P value <0.05.

In terms of CV death, both the combined use and individual use of SGLT2i or Pio showed lower risks compared with non-study drugs (combined: aHR, 0.54; 95% CI, 0.38–0.77; SGLT2i: aHR, 0.55; 95% CI, 0.44–0.68; Pio: aHR, 0.84; 95% CI, 0.73–0.98, respectively). The combined use of SGLT2i and Pio also exhibited the lowest risk of CV death compared with non-study drugs (aHR, 0.54; 95% CI, 0.38–0.77) (**Figure 2B**).

Regarding non-fatal MI, no significant differences were observed among the four groups (**Figure 2C**). Regarding non-fatal ischemic stroke, both the combined use and the individual use of SGLT2i or Pio were associated with lower risks of non-fatal ischemic stroke compared with non-study drugs (combined: adjusted subdistribution hazard ratio [aSHR], 0.70; 95% CI, 0.53–0.92; SGLT2i: aSHR, 0.82; 95% CI, 0.69–0.97; Pio: aSHR, 0.76; 95% CI, 0.65–0.89, respectively). The combined use of SGLT2i and Pio also exhibited the lowest risk of non-fatal ischemic stroke compared with non-study drugs (aSHR, 0.70; 95% CI, 0.53–0.92) (**Figure 2D**).

In terms of hospitalization for HF, the use of SGLT2i alone was associated with a significantly lower risk of hospitalization for HF compared with non-study drugs (aSHR, 0.77, 95% CI 0.60–0.97). There was no significant difference in the risk of hospitalization for HF with the use of Pio alone (aSHR, 0.82; 95% CI, 0.67–1.01) or the combined treatment (aSHR, 0.73; 95% CI, 0.49–1.07) compared with non-study drugs (**Figure 2E**).

### Effects of SGLT2i and Pio on all-cause mortality in T2DM patients

**Figure 3:**
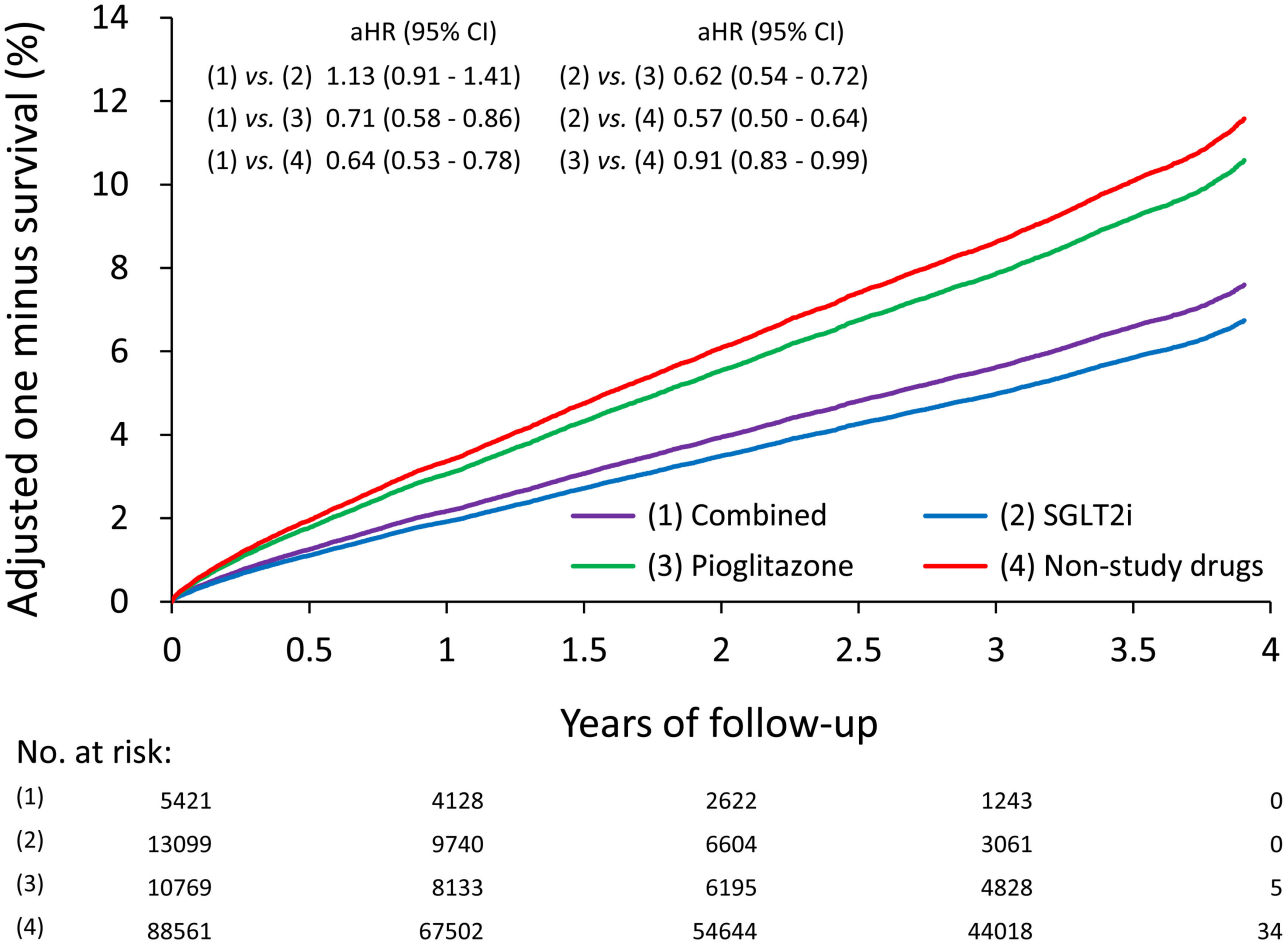
The adjusted/fitted (by multiple propensity scores) one minus survival rates of all-cause mortality of T2DM patients according to the use of SGLT2i and Pio.

The results showed that both the combined use and the individual use of SGLT2i or Pio were associated with lower risks of all-cause mortality compared with non-study drugs (combined: aHR, 0.64; 95% CI, 0.53–0.78; SGLT2i: aHR, 0.57; 95% CI, 0.50–0.64; Pio: aHR: 0.91; 95% CI, 0.83–0.99, respectively) (**Table 3**; **Figure 3**).

**Table 3:** Secondary outcomes of patients according to the use of SGLT2i and pioglitazone.

Outcome/group	Incidence† (95% CI)	Adjusted HR or SHR (95% CI) (column vs row)			Adjusted *P* value of trend test
		Combined	SGLT2i	Pioglitazone	
All-cause death					<0.001
Non-study drugs	36.7 (35.9–37.5)	0.64 (0.53–0.78)*	0.57 (0.50–0.64)*	0.91 (0.83–0.99)*	
Pioglitazone	24.4 (22.5–26.4)	0.71 (0.58–0.86)*	0.62 (0.54–0.72)*		
SGLT2i	10.9 (9.7–12.2)	1.13 (0.91–1.41)			
Combined	11.1 (9.0–13.1)	–	–	–	
DKA or HHS					<0.001
Non-study drugs	176.5 (174.4–178.6)	1.07 (1.02–1.13)*	1.21 (1.17–1.26)*	1.03 (0.99–1.07)	
Pioglitazone	205.9 (199.2–212.6)	1.05 (0.99–1.11)	1.18 (1.13–1.24)*		
SGLT2i	300.4 (291.9–308.9)	0.88 (0.84–0.94)*			
Combined	269.2 (257.1– 281.4)	–	–	–	
Hypoglycemia with glucose<55 mg/dL					0.001
Non-study drugs	5.7 (5.4–6.0)	0.90 (0.69–1.17)	1.08 (0.91–1.29)	1.01 (0.86–1.19)	
Pioglitazone	8.4 (7.3–9.6)	0.89 (0.67–1.18)	1.07 (0.87–1.31)		
SGLT2i	7.5 (6.4–8.6)	0.83 (0.63–1.10)			
Combined	6.6 (5.0–8.2)	–	–	–	
Newly diagnosed malignancy					0.080
Non-study drugs	14.3 (13.8–14.8)	0.85 (0.69–1.06)	0.92 (0.81–1.06)	0.96 (0.85–1.08)	
Pioglitazone	12.5 (11.1–13.9)	0.89 (0.71–1.13)	0.97 (0.82–1.14)		
SGLT2i	11.0 (9.7–12.3)	0.92 (0.73–1.17)			
Combined	9.8 (7.9–11.7)	–	–	–	
Acute hepatitis					0.666
Non-study drugs	1.00 (0.87–1.13)	0.73 (0.25–2.12)	0.92 (0.55–1.52)	1.16 (0.72–1.88)	
Pioglitazone	0.71 (0.38–1.04)	0.63 (0.20–1.95)	0.79 (0.41–1.52)		
SGLT2i	0.79 (0.45–1.14)	0.80 (0.26–2.47)			
Combined	0.38 (0.01–0.76)	–	–	–	

SGLT2i, sodium-glucose co-transporter-2 inhibitors; HR, hazard ratio; SHR, sub-distribution hazard ratio; DKA, diabetic ketoacidosis; HHS, hyperosmolarhyperglycemia.

†Number of events per 1000 person-years.

*P value <0.05.

### Effects of SGLT2i and Pio on major side effects in T2DM patients

The combined use of SGLT2i and Pio or the use of SGLT2i alone carried higher risks of DKA/HHS compared with non-study drugs (combined: aSHR: 1.07; 95% CI, 1.02–1.13; SGLT2i: aSHR, 1.21; 95% CI, 1.17–1.26). The use of SGLT2i alone had a higher risk of DKA/HHS compared with the use of Pio alone (aSHR, 1.18; 95% CI, 1.13–1.24). No significant differences in risks were noted among the four groups for hypoglycemia, newly diagnosed malignancy, or acute hepatitis (**Table 3**).

### Subgroup analysis

**Figure 4:**
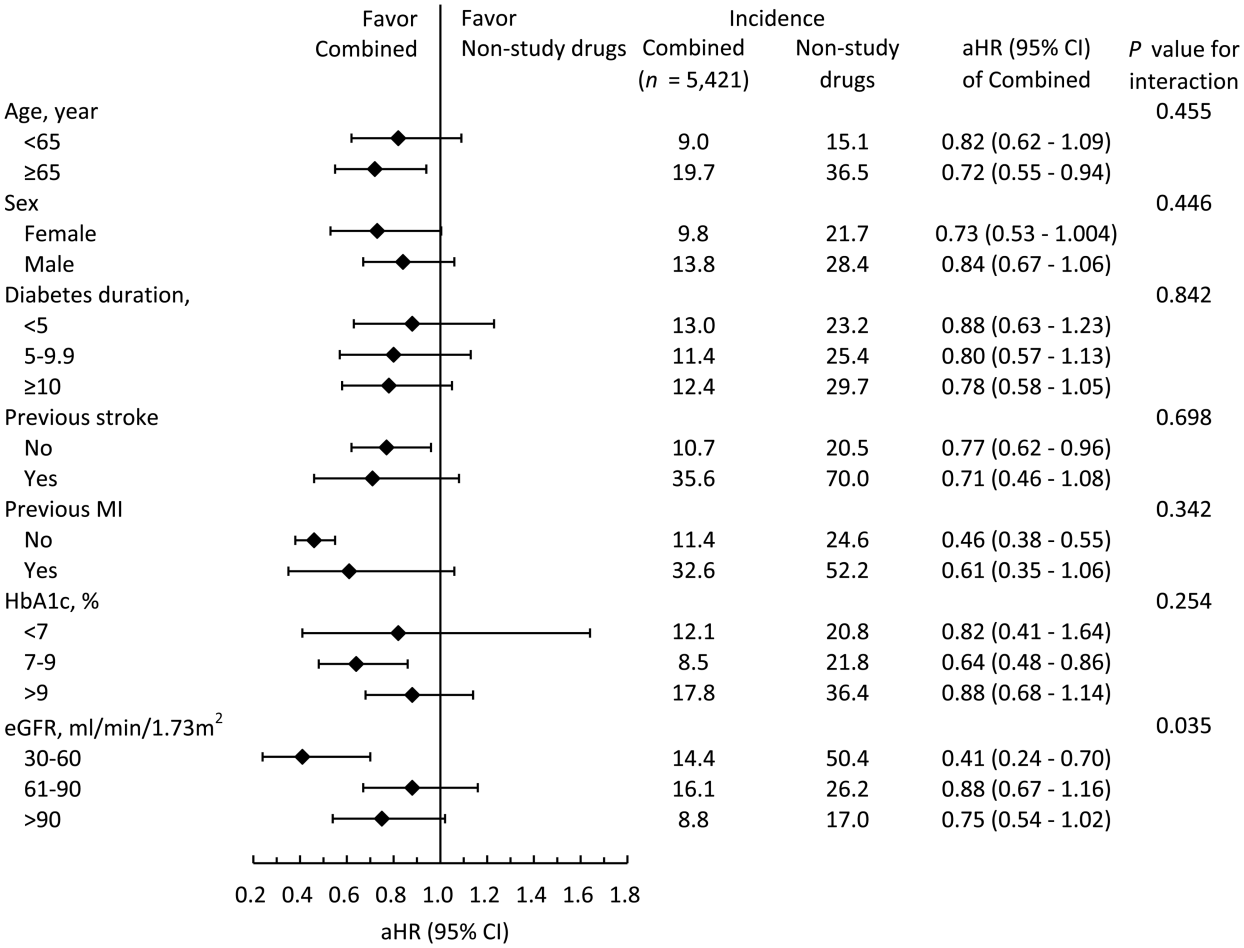
The subgroup analysis of the risks of 4P-MACE between the combined drugs group and non-study drugs group.

Stratified analyses were conducted to verify the consistent effects of combined treatment versus non-study drugs on the risk of 4P-MACE across subgroups, including age, sex, DM duration, previous stroke, previous MI, presence of CKD, HbA1c level, and eGFR. The effects were generally consistent in favor of combined treatment of SGLT2i and Pio versus non-study drugs (**Figure 4**). It appeared that patients with eGFR between 30-60 ml/min/1.73m2 benefited more from the protective effect of combined treatment on 4P-MACE (aSHR: 0.41, 0.88, and 0.75 in eGFR categories of 30-60, 61-90, and >90 ml/min/1.73m2, respectively; *P* for interaction = 0.035).

## Discussion

In this retrospectively observational cohort study from the largest multi-institutional databank in Taiwan, we demonstrated that combined treatment of SGLT2i and Pio was associated with the decreased risks of 4P-MACE and all-cause mortality compared with those who did not take SGLT2i and Pio during a mean follow-up for 2.2 years. Besides, regarding the individual component of the primary outcomes, the group of combined treatment with SGLT2i and Pio had the lowest risks of CV death (aHR, 0.54; 95% CI, 0.38–0.77) and non-fatal ischemic stroke (aSHR, 0.70; 95% CI, 0.53–0.92) compared with non-study drugs group. Moreover, addressing the apprehensions regarding potential HF risk associated with Pio prescription, our study found no discernible rise in the risk of HF when SGLT2i was combined with Pio treatment. The side effects of Pio on HF may be alleviated by SGLT2i. Although our study excluded T2DM patients with HF, there are still some patients with history of CAD, old MI, old stroke, PAD, HTN, dyslipidemia, and CKD. Therefore, the CV outcomes in our study cohort consisted of primary prevention for patients with multiple risks and secondary prevention.

The synergistic effects observed in our study with the combined treatment of SGLT2i and Pio are supported by meta-analysis studies demonstrating the individual CV benefits of both medications across diverse subsets of patients with T2DM (23–28). Specifically, SGLT2i have shown strengths in renal protection and prevention of hospitalization for HF in patients with multiple risk factors for T2DM (28, 29). Additionally, in patients with established CVD, SGLT2i have demonstrated renal protection, prevention of hospitalization for HF, and reduction in major adverse CV events. However, it’s noteworthy that in the EMPA-REG trial, the risk of stroke after treatment with empagliflozin increased nonsignificantly (5, 29, 30).

On the other hand, the strengths of Pio for T2DM include its metabolic regulatory effects, neuroprotective properties, and its ability to ameliorate the progression of atherosclerosis owing to its potent role as a PPAR-γ agonist. Studies have suggested that Pio may reduce the risks of stroke, non-fatal MI, and all-cause mortality. However, Pio is also associated with increased risks of HF, particularly in patients with CKD, as CKD is a major predictor of hospitalization for HF (12–14, 31–34). Therefore, the combined use of SGLT2i and Pio complements each other. Pio can improve T2DM patients who take SGLT2i to decrease risks of stroke, and SGLT2i can improve T2DM patients who take Pio to decrease risks of HF, especially in CKD patients. Our results showed that patients with eGFR between 30-60 ml/min/1.73m2 benefited more from the protective effect of combined treatment on 4P-MACE (aSHR: 0.41, 0.88, and 0.75 in eGFR categories of 30-60, 61-90, and >90 ml/min/1.73m2, respectively; *P* for interaction = 0.035). Our subgroup analysis further supported this theory, highlighting the potential of combined therapy to address the multifaceted CV risks in T2DM patients. In this new era of SGLT2i, Pio, this old drug, has found a new role in the treatment of T2DM and is a cost-effective medication which makes it a valuable addition to the treatment armamentarium for T2DM patients. Its affordability further enhances its appeal, especially in resource-limited settings where access to expensive medications may be challenging.

Regarding safety outcomes, the combined treatment of SGLT2i and Pio was not associated with a higher risk of hypoglycemia, newly diagnosed malignancy, or acute hepatitis. However, it was found to mildly increase the risks of DKA/HHS.

### Limitations

In our study, there are several limitations that require careful consideration. Firstly, this is a retrospective analysis although it was conducted using a large-scale database. In order to study a causal relationship, a well-designed and randomized trial is warranted. Secondly, the diagnoses in our cohort study are based on ICD-9 or ICD-10 codes from insurance reimbursement records. The fidelity of our data relies on the precision and comprehensiveness of the initial records. Furthermore, given the observational nature of the study design, it’s important to acknowledge the possibility of unmeasured confounders that could impact the observed associations. Thirdly, drug adherence could have influenced the study results, and in a real-world study, we can only presume drug adherence based on prescription records. Besides, only three kinds of SGLT2i (empagliflozin, dapagliflozin, and canagliflozin) were included in our cohort study. Moreover, performing head-to-head propensity score matching in this study is not feasible because there will be six sets of matchings. The inferential population would be not clear due to the sample size of one study group (e.g., combined use of SGLT2i and Pio) can be different in each set of propensity score matching when compared to other three study groups. Lastly, this study encompasses only Asian patients in Taiwan, and the outcomes may not be readily extrapolated to the broader population or diverse ethnic groups.

## Conclusions

In T2DM patients without HF, the combined treatment with SGLT2i and Pio may synergistically provide CV benefits without increasing risks of HF. The combination of SGLT2i and Pio represents not only a clinically effective strategy but also a financially prudent approach to managing T2DM and reducing CV risk.

## Data Availability

The raw data supporting the conclusions of this article will be made available by the authors, without undue reservation.
